# The Effects of Maltreatment in Childhood on Working Memory Capacity in Adulthood

**DOI:** 10.5964/ejop.v13i4.1373

**Published:** 2017-11-30

**Authors:** Arta Dodaj, Marijana Krajina, Kristina Sesar, Nataša Šimić

**Affiliations:** a Department of Psychology, University of Zadar, Zadar, Croatia; b Department of Psychology, University of Mostar, Mostar, Bosnia and Herzegovina; c Centre of Mental Health, Široki Brijeg, Bosnia and Herzegovina; d Department of Psychology, University of Zadar, Zadar, Croatia; Department of Psychology, Webster University Geneva, Geneva, Switzerland; University of South Wales, Newport, United Kingdom

**Keywords:** maltreatment in childhood, trauma, cognitive functioning, working memory, executive functions

## Abstract

The aim of this study was to research the relation between exposure to maltreatment in childhood and working memory capacity in adulthood. A survey among 376 females in the age between 16 and 67 was administered. Exposure to maltreatment in childhood (sexual, physical and psychological abuse, neglect and witnessing family violence) was assessed retrospectively using the Child Maltreatment Questionnaire (Karlović, Buljan-Flander, & Vranić, 2001), whilst the Working Memory Questionnaire (Vallat-Azouvi, Pradat-Diehl, & Azouvi, 2012) was used to assess working memory capacity (recalling verbal information, numerical information, attention ability and executive functioning). The results suggest a significantly greater prevalence of physical abuse and witnessing family violence in comparison to other forms of maltreatment in childhood. Psychological abuse and witnessing family violence have shown themselves to be statistically significant predictors for deficits in total working memory capacity, verbal recall and attention ability. The results suggest that traumatic experiences during childhood, such as abuse, may trigger particular cognitive changes which may be reflected in adulthood. It is, therefore, exceedingly important to conduct further research in order to contribute to the understanding of the correlation between cognitive difficulties and maltreatment in childhood.

Child abuse and neglect are understood to be the act and/or the failure to act of a parent or caregiver which results in harm, potential harm or a threat to a child, even in situations in which the harm is accidental or unintentional (Gilbert et al., 2009; Hart & Rubia, 2012). The different forms of abuse and neglect are most frequently categorised as physical abuse, psychological abuse, neglect, sexual abuse and witnessing family violence (Higgins, 2004). According to the results of a meta-analytical study (Stoltenborgh, Bakermans-Kranenburg, Alink, & van Ijzendoorn, 2015) 36% of the subjects had been exposed to psychological abuse, 23% to physical abuse, 18% to emotional neglect, 16% to physical neglect and 13% to sexual abuse.

The exposure to abuse and neglect in childhood is a serious stress, which, besides emotional and behavioural difficulties, can also lead to cognitive deficits (Hart & Rubia, 2012). The studies which have researched the relationship between child abuse and specific cognitive deficits in adulthood (Hart & Rubia, 2012) suggest the detrimental effect of abuse and neglect on short-term memory capacity, episodic verbal memory, working memory, speech and language abilities, planning and organisational abilities, executive functioning, and abilities to direct and sustain attention, difficulties in learning and remembering (Irigaray et al., 2013; McCrory, De Brito, & Viding, 2010). Moreover, abused individuals achieve poorer results on general cognitive ability tests (McCrory et al., 2010).

Studies which have researched the connection between specific forms of abuse and neglect in childhood and cognitive functioning in adulthood suggest that individuals exposed to neglect, physical and sexual abuse have significantly poorer verbal memory capacity in adulthood than do non-abused individuals (Grassi-Oliveira, Ashy, & Stein, 2008). Neglect, as opposed to other forms of abuse, has a more significant influence on difficulties in executive functioning according to the results of a longitudinal study by Nikulina and Widom (2013). The relationship between psychological abuse, neglect and difficulties in numerical memory capacity was confirmed in a study by Majer et al. (2010), while the effect of other forms of maltreatment on numerical memory capacity was nonsignificant. In contrast to the previously mentioned study results, Gould et al. (2012) indicated that difficulties in executive functioning were more significantly connected to physical and psychological abuse and neglect, whilst difficulties in numerical memory were more significantly linked to sexual abuse.

Although the results of the previously mentioned studies indicate a connection between child maltreatment and deficit in cognitive abilities in adulthood, they do not offer any proof that the indicated deficits have a significant effect on the daily functioning of the individuals who have been evidenced as such. Furthermore, the studies to date have assessed exposure to one or two forms of maltreatment in childhood or they have been directed at maltreatment in general, or the forms of maltreatment were not specified at all (Hart & Rubia, 2012). The aforementioned lack has motivated us to attempt to determine the difficulties in daily functioning, which are connected to different forms of maltreatment, and to attempt to establish which difficulties are most frequently connected with specific forms of maltreatment in childhood. Thus, the aim of this study was to determine the relationship between different forms of child maltreatment (physical and psychological abuse, neglect and witnessing family violence) and working memory difficulties (working memory capacity for verbal and numeric information, executive functioning and attention ability), and hence determine the predictive value of different forms of maltreatment on different memory types.

## Methods

### Participants

The study sample consisted of 376 females aged from 16 to 67 years of age, of which 142 women had been exposed to different forms of maltreatment in childhood (*Mdn* = 2.517, *IQR* = 1.000), and 234 women had not been exposed to maltreatment in childhood (*Mdn* = 1.980, *IQR* = .767).

### Measures

#### Working Memory Capacity

In order to assess working memory capacity, the Working Memory Questionnaire by Vallat-Azouvi et al. (2012) was used. The Working Memory Questionnaire was developed to estimate everyday life problems related to deficits of working memory in brain injured patients. The Questionnaire was designed using Baddeley’s memory model, which suggests that the concept of working memory does not only refer to short-term memory but also incorporates attention and executive functioning abilities (Vallat-Azouvi et al., 2012). The Questionnaire serves to assess four types of difficulties in working memory: difficulties in recalling verbal information, difficulties in recalling numerical information, difficulties in executive functioning and difficulties in attention ability. It consists of 30 questions where participant rated each question on a five-point Likert-type scale, ranging from 0 (“no problem at all”) to 4 (“very severe problem in everyday life”). The statement content on the subscale of difficulties in working memory recall of verbal information is concerned with difficulties in textual comprehension (e.g., “Do you have difficulty understanding what you read?”) and the retention of verbal information (e.g., “Do you find it difficult to remember the name of a person who has just been introduced to you?”). The statements on the subscale of difficulties in working memory recall of numerical information are concerned with difficulties in mental processing and recalling numerical information (e.g., “When you pay cash for an item, do you have difficulty in realizing if you have been given the correct change?”, “Do you have problems with remembering sequences of numbers, for example, when you have to note down a telephone number?”). The statements on the subscale of difficulties in executive functioning are concerned with difficulties in organizing, planning and making decisions (e.g., “Do you feel that you are very slow to carry out your usual activities” and “Do you have difficulty in organizing your time with regard to appointments and your daily activities?”). Finally, the statements on the subscale of difficulty in attention ability are concerned with difficulties in focusing and the inability to concentrate (e.g., “Do nearby conversations disturb you during a conversation with another person?”), and with the difficulty of doing activities in chronological order (e.g., “Do you find it difficult to carry out an activity with chronological steps (cooking, sewing, DIY)?”). The total result on a specific subscale was gained through the simple addition of participant assessment. The range of results for each of the subscales was from 10 to 50, and from 30 to 150 for the whole questionnaire. Higher scores corresponded to more difficulties/complaints.

#### Maltreatment in Childhood

The exposure to physical and psychological abuse in childhood, neglect and witnessing family violence was assessed with the Child Maltreatment Questionnaire (Karlović, Buljan-Flander & Vranić, 2001) based on the Comprehensive Child Maltreatment Scales for Adults (Higgins & McCabe, 2001a). The participants were requested to record how often, up to the age of 14, they were exposed to the following behaviour by their father, mother or any other older person from their environment. This allows their comparison and reveals whether the child was abused by one or more individuals.

The questions for the assessment of psychological abuse included the following behaviours: yelling, making fun of and mocking, talking dirty, frightening and threats, callous criticism and comparisons, insults and overly forbidding. The content of the question on physical abuse included the following behaviours: slapping and thumping, beatings, punching or hitting with an object, throwing on the ground and grievous physical injury. The content of the question on neglect included the following parental behaviour: irregular provision of meals and clothing, not visiting the doctor on time, locking up alone for longer periods of time, ignoring and refusing to talk. The content of the question for the assessment of witnessing family violence included the following behaviours: criticizing, insulting, threatening, limiting and yelling at other family members, as well as beating, hitting and injuring other family members. The participants rated every statement, which described the potentially abusive behaviour of parents or other adults towards them, with a three-point scale, ranging from 1 (never) to 3 (often).

On the basis of the participants’ answers, the total result of the participants for every question was obtained through simple addition, as was the total result for the whole scale. In addition, whilst calculating the exposure to child maltreatment, dichotomous results were used, i.e., the participants were divided into two categories: the abused and the non-abused. In accordance with studies to date, the results on the specific subscales of maltreatment above a 75 percentile was used to categorize exposure to maltreatment in childhood (Holt & Espelage, 2007). Therefore, the participants whose results on the subscales were in the upper 25 percentile band were categorized as having been exposed to a specific form of child maltreatment. Due to the set problem in determining exposure to maltreatment, we decided to also collate the results using the dichotomous method.

### Procedure

The questionnaire was conducted on-line, via the web-tool Survey Monkey. The questionnaires were distributed to the participants in two ways: via social networks or via e-mail addresses, simultaneously informing them about the research being conducted. The participants were informed of the aims of the research and the anonymity of all data was stressed. It was also stressed that the assessment is on a volunteer basis and that they could withdraw from the assessment at any time. The participants were requested to be as honest as possible, and they were cautioned that there are no correct or incorrect answers. The participants filled out the questionnaires in a set order which was determined in advance: first, they had to fill out a form with questions concerning general information on the participant, then they filled the working memory questionnaire, and finally the retrospective self-assessment questionnaire on exposure to child maltreatment. The participants needed approximately 15 minutes to fill out the questionnaires.

## Results

The results for the statements in the retrospective assessment of child maltreatment indicate that 142 (31.63%) participants of the total sample (*N* = 376) were exposed to one or more forms of child maltreatment (Table 1). Physical abuse (20.04%) and witnessing family violence (18.26%) are the most common forms of child maltreatment, while psychological abuse (11.36%) and neglect (7.8%) are present to a significantly lesser degree. Besides the frequency assessment, Table 1 also depicts the basic descriptive parameters or mean values (*Mdn*) and measured variability (*Q1-Q3*) for each type of maltreatment. The resulting values indicate an on-average greater prevalence of witnessing family violence and psychological abuse in comparison to other forms of maltreatment.

**Table 1:** Descriptive Parameters of Prevalence of Different Form of Maltreatment in Childhood by Participants Exposed and Non Exposed to Abuse

Form of maltreatment in childhood^a^	Exposed to abuse			Non-exposed to abuse			Total	
	*n* (%)	*Mdn*	*Q1-Q3*	*n* (%)	*Mdn*	*Q1-Q3*	*Mdn*	*Q1-Q3*
Psychological abuse	51 (13.56)	2.000	.333	325 (86.44)	1.000	.333	1.333	.667
Physical abuse	90 (23.94)	1.667	.333	286 (76.06)	1.000	.000	1.000	.000
Neglect	35 (9.31)	1.500	.500	341 (90.69)	1.000	.000	0.000	.000
Witnessing family abuse	82 (21.81)	5.000	1.000	294 (78.19)	3.000	.000	3.000	1.000

The differences in working memory capacity with respect to exposure to different types of child maltreatment were tested with the Mann-Whitney U test (Table 2). The participants who had been exposed to psychological abuse in childhood, in comparison to those participants who had never experienced psychological abuse, exhibited greater difficulties in all aspects of working memory (verbal, numerical, executive functioning, attention ability), with the difference being statistically significant. The most significant differences were determined in the total result on the working memory scale (*Z* = 5.267, *p* < .001) and in attention abilities (*Z* = 5.010, *p* < .001). Lower, but nevertheless significant differences, were evidenced in verbal information recall (*Z* = 4.948, *p* < .001), executive functioning (*Z* = 4.642, *p* < .001) and numerical information recall (*Z* = 4.132, *p* < .001). In order to enable a clearer comparison of the attained results and to analyse the possible implications thereof, effect sizes were also determined using the Cohen d-index (Table 2). The resulting differences between the participants who had been subject to psychological abuse and those who had not, according to the results obtained using the Mann-Whitney U test, were moderate to high on all the subscales of working memory capacity. The highest effect size obtained was in the difficulties in the total working memory capacity (*d* = .814) and in verbal information recall (*d* = .803) amongst the participants who had experienced psychological abuse and those who had not, whilst difficulties in executive functioning (*d* = .737), attention ability (*d* = .711) and numerical information recall (*d* = .589) showed a lower or average effect size.

**Table 2:** Descriptive Parameters and Testing Differences Between Difficulties in Working Memory Capacity With Regards to With Respect to Exposure to Different Types of Maltreatment in Childhood

	Psychological abuse						Physical abuse						Neglect						Witnessing family abuse					
	Exposed to abuse		Non-exposed to abuse				Exposed to abuse		Non-exposed to abuse				Exposed to abuse		Non-exposed to abuse				Exposed to abuse		Non-exposed to abuse			
Working memory capacity	*Mdn*	*IQR*	*Mdn*	*IQR*	*Z*	*d*	*Mdn*	*IQR*	*Mdn*	*IQR*	*Z*	*d*	*Mdn*	*IQR*	*Mdn*	*IQR*	*Z*	*d*	*Mdn*	*IQR*	*Mdn*	*IQR*	*Z*	*d*
Verbal memory	2.549	1.125	1.972	1.750	4.948**	.803	2.125	1.263	1.955	.875	3.942**	.491	2.554	1.750	1.999	1.000	3.446**	.654	2.415	1.263	1.949	.535	4.277**	.565
Numerical memory	2.340	1.000	1.868	1.000	4.132**	.589	2.198	1.333	1.849	.833	3.374**	.428	2.451	1.500	1.879	1.000	3.592**	.662	2.203	1.500	1.875	.883	2.811*	.412
Executive functioning	2.989	1.571	2.370	1.143	4.642**	.737	2.834	1.429	2.334	1.000	4.567**	.584	2.968	1.000	2.401	1.143	4.004**	.708	2.845	1.286	2.344	1.143	4.559**	.608
Attention	2.239	1.038	1.758	.667	5.010**	.711	2.051	1.250	1.752	.667	3.168*	.423	2.273	1.038	1.778	.667	3.518**	.364	2.180	1.333	1.724	.667	4.053**	.659
Total working memory	2.517	1.000	1.980	.767	5.267**	.814	2.344	1.267	1.961	.754	4.305**	.777	2.546	1.100	2.002	.800	4.231**	.777	2.402	1.145	1.955	.733	4.667**	.643

*p < .01. **p < .001.

Statistically significant differences were determined for all types of difficulties in working memory capacity between the participants who had been physically abused in childhood and non-abused participants (Table 2). The highest significant differences were obtained for difficulties in executive functioning (*Z* = 4.567, *p* < .001) and in total working memory capacity (*Z* = 4.305, *p* < .001), while the lowest significant differences were obtained for difficulties in attention maintenance (*Z* = 3.168, *p* < .01). The results of the Cohen d-index point in the same

direction, although to a moderate to a moderate to moderately-large effect size. The highest significant difference value between the physically abused and the non-abused participants is evident in the difficulties in total working memory capacity (*d* = .777) and executive functioning (*d* = .584), whereas the lowest was evident in the difficulties in recalling numerical information (*d* = .428) and in maintaining attention (*d* = .423).

Further analysis of the results, between the participants who had been maltreated in childhood and those who had not, indicates that there is a significant difference between the two groups in all the measures of working memory capacity (Table 2). The most significant differences were obtained on the subscales of total working memory capacity (*Z* = 4.231, *p* < .001) and executive functioning (*Z* = 4.004, *p* < .01), whilst the least differences were ascertained on the subscale of verbal information recall (*Z* = 3.446, *p* < .001). Table 2 clearly illustrates that the values of the effect sizes indicate a similar direction. The greatest difference between the two groups is evident on the subscale of total working memory capacity (*d* = .777) and executive functioning (*d* = .708), which indicates an exceedingly high effect, whilst the smallest difference was evident on the subscale of attention ability (*d* = .364), which indicates a moderate effect.

The final analysis addressed the difficulties in working memory capacity with respect to the participants’ exposure or non-exposure to family violence (Table 2). Statistically significant differences were ascertained on all the subscales of working memory capacity between those participants who had witnessed family violence and those who had not. The most significant differences were ascertained for total working memory capacity (*Z* = 4.667, *p* < .001) and executive functioning (*Z* = 4.559, *p* < .001), and the least significant differences for numerical information recall (*Z* = 2.811, *p* < .001). If the obtained differences are compared to the values of the Cohen d-index, it is evident that the average effect size is exhibited on the subscale of attention ability (*d* = .659), total working memory capacity (*d* = .643) and on the subscale of executive functioning (*d* = 608), whereas the lowest, or weakest size is evident on the subscale of numerical information recall (*d* = .412).

In order to reveal whether a relationship exists between child maltreatment and difficulties in working memory capacity, Spearman’s rank correlation analysis was applied. The main reason for the application of Spearman’s rank correlation was the asymmetrical distribution of the result variables of maltreatment in childhood and the variables of working memory capacity. On the basis of the obtained results (Table 3), we can conclude that lower statistically significant correlations have been determined between all forms of maltreatment and neglect in childhood with difficulties in working memory capacity. Higher correlations are evident on the subscales of total working memory capacity, attention ability and verbal information recall with all the tested forms of maltreatment, and lower correlations on the subscale of numerical information recall. Similarly, a higher correlation was ascertained between psychological abuse and witnessing family violence and all aspects of working memory capacity in comparison to physical abuse and neglect, where these correlations are somewhat lower.

**Table 3:** Correlations Between Forms of Maltreatment in Childhood and Difficulties in Working Memory Capacity

Working memory	Forms of maltreatment in childhood			Witnessing family abuse
	Psychological abuse	Physical abuse	Neglect	
Verbal memory	.307**	.222**	.201**	.321**
Numerical memory	.229**	.189**	.200**	.247**
Executive functioning	.352**	.277**	.189**	.288**
Attention	.306**	.213**	.211**	.336**
Total working memory	.349**	.262**	.232**	.349**

*p < .01. **p < .001.

In order to test the predictive value of various forms of maltreatment for difficulties in working memory capacity, multiple regression analysis was applied. The analyses were conducted thereby that the criteria variable in every individual, conducted analysis was a type of working memory capacity (difficulties in verbal information recall, difficulties in numerical information recall, difficulties in executive functioning and difficulties in attention capacity), and the predictor variables were the forms of maltreatment (psychological, physical, neglect, witnessing family violence). According to the criteria set out by Myers (1990), before conducting regression analysis, it is necessary to determine the variance inflation factor (VIF) in order to determine the non-existence of multi-collinearity between models. Variance inflation factors are determined thus: first, the tolerance is calculated for every working memory capacity subscale, after which the value of 1 is divided by the tolerance for every type of working memory. The determined VIF for every predictor in every model is less than 10, which indicates the non-existence of multi-collinearity between the models, which satisfies the precondition for the non-existence of multi-collinearity before the introduction of the regression analysis. The significant regression model predictors for working memory capacity for different types of child maltreatment are shown in Table 4.

**Table 4:** Predictive Value of Various Forms of Maltreatment in Childhood and Neglect for Difficulties in Working Memory Capacity

Criterion	Predictor	*R*^2^	β	*F*	*df*	*p*
Difficulties in verbal memory		.119		12.582	4	
	Psychological abuse		.162			.006
	Physical abuse		.011			.850
	Neglect		.065			.207
	Witnessing family abuse		.170			.007
Difficulties in numerical memory		.077		7.703	4	
	Psychological abuse		.104			.081
	Physical abuse		.035			.557
	Neglect		.099			.060
	Witnessing family abuse		.107			.097
Difficulties in executive functioning		.133		14.238	4	
	Psychological abuse		.224			.000
	Physical abuse		.090			.117
	Neglect		.058			.253
	Witnessing family abuse		.064			.305
Difficulties in attention		.126		13.391	4	
	Psychological abuse		.156			.007
	Physical abuse		-.010			.868
	Neglect		.071			.167
	Witnessing family abuse		.195			.002
Total working memory		.150		16.335	4	
	Psychological abuse		.189			.001
	Physical abuse			.035		.540
	Neglect		.083			.100
	Witnessing family abuse		.159			.011

*Note*. *R* = coefficient of multiple correlation. *R^2^* = coefficient of multiple determination (percent of criteria variance explained by predictors). β = beta coefficients of examined. *df* = degrees of freedom.

For the difficulties in verbal information recall amongst the abused participants, significant predictors proved to be the variables of psychological abuse and witnessing family violence, which combined account for approximately 12% variance of verbal information recall. The positive beta ponder indicates that frequent exposure to psychological abuse and frequent witnessing of family violence predicts more significant difficulties in verbal information recall.

For difficulties in numerical information recall amongst the abused participants, not a single predictor variable proved to be significant.

Psychological abuse proved to be a determinant for the difficulties in executive functioning, accounting for approximately 13% variance of the difficulties in executive functioning. The obtained values of the beta ponder indicate that frequent exposure to psychological abuse suggests a greater probability of developing difficulties in executive functioning.

The results of the regression analysis conducted on the subscale of attention ability showed the variables of psychological abuse and witnessing family violence to be a significant predictor variable. The variance percentage of difficulties in attention ability which these two types of maltreatment suggest is relatively low and amounts to approximately 13%. The beta ponders indicate that there exists a greater probability that persons who were frequently exposed to psychological abuse and witnessed family violence will have more express difficulties with attention maintenance.

The final regression analysis confirmed that the significant determinants for difficulties in total working memory capacity are psychological abuse and witnessing family violence. The positive beta ponders indicate that frequent exposure to psychological abuse, neglect and witnessing family violence predicts a greater probability of difficulties with total working memory capacity. The obtained significant predictors allow for 15% variance of difficulties in total working memory capacity.

## Discussion

The results of the conducted research indicate that persons who had been exposed to some form of maltreatment in childhood have significantly more difficulties in working memory capacity than do persons who have not experienced maltreatment. Psychological abuse is the form of abuse which leads to the greatest negative consequences on cognitive functioning amongst abused individuals. The consequences are most significant in the field of general working memory capacity and executive functioning.

The most frequent form of child maltreatment that the participants had been exposed to were physical abuse and witnessing family violence, which is in accordance with other studies conducted in Croatia and Bosnia and Herzegovina (Ćosić, Buljan Flander, & Karlović, 2002; Sesar, Živčić-Bečirević, & Sesar, 2008). In contrast to the aforementioned studies, the results of studies conducted in the Germany emphasize of a higher prevalence rates of physical neglect, then emotional neglect, whereas sexual and psychological abuse are less prevalent (Iffland et al., 2013). One of the possible explanations is that violence in our sample is perceived solely as a behaviour which is manifested physically. All other types of violence, which are physically less visible, may be perceived as usual and acceptable. Such a behavioural model is frequently a reflection of culturological values acquired in the primary environment. Another possible explanation for the difference in the study results may also be found in the methodological difference in the assessment of maltreatment frequency. The data in this study was collected on the basis of retrospective reports on maltreatment in childhood. The data collected are of a private and intimate nature, requiring the participants to recall negative childhood experiences. Some of the negative experiences may have been repressed and/or forgotten, while others may have been exaggerated. Furthermore, the negative emotions connected to recalling traumatic experiences may have influenced the ability of the participant to critically evaluate the behaviour of her parents (Higgins & McCabe, 2001b). Finally, Finkelhor (1994) claims that all assessment obtained through retrospective research is practically always underestimated.

The results of this study coincide with the results of other studies conducted to date (Gould et al., 2012; Grassi-Oliveira et al., 2008; Hart & Rubia, 2012; Irigaray et al., 2013; Majer et al., 2010; McCrory et al., 2010; Nikulina & Widom, 2013) which indicated significant differences in all subscales of working memory capacity between abused and non-abused participants. More significant difficulties other than in total working memory capacity have been indicated for executive functioning amongst persons who have been exposed to physical abuse, neglect and witnessed family violence. On the other hand, those who had been psychologically abused exhibited, besides difficulties in working memory capacity, significant difficulties in maintaining attention and verbal information recall. The obtained results show the most highlighted effects of maltreatment to be on executive functioning. The difficulties in the mentioned cognitive domains may be explained through the activation of the hypothalamic–pituitary–adrenal (HPA) axis, as well as by the changes in the prefrontal and anterior cingulate cortex which are the basis for the control of working memory function and executive functioning. Namely, exposure to maltreatment in childhood significantly affects the higher secretion of the norepinephrine, which increases the activities of the HPA axis. The HPA axis then increases the secretion of the adrenocorticotropic hormone which consequently provokes neural changes in the prefrontal and anterior cingulate cortex (De Bellis, 2005; Grassi-Oliveira et al., 2008; Panzer, 2008; Watts-English, Fortson, Gibler, Hooper, & De Bellis, 2006). Existing evidence which indicates neurodegeneration in the prefrontal and anterior cingulate cortex may also contribute to the mentioned difficulties evoked by maltreatment (Anderson et al., 2008 as cited in Hart & Rubia, 2012; Bremner et al., 1999; De Bellis et al., 2002; Hanson et al., 2012; Hart & Rubia, 2012). Furthermore, as executive functions serve to optimize execution in situations which demand the coordination of several cognitive processes, including working memory and attention, their biological foundation is thus more complex than individual cognitive functions. The importance of the relationship between the dorsolateral prefrontal cortex and executive functions must be addressed, as this is the region involved in working memory. Therefore, it is believed that working memory plays the central role in executive functioning (Banich & Compton, 2011), which could explain the obtained results on the most evident effects of all types of maltreatment and neglect on executive functioning.

Furthermore, psychological abuse has the greatest consequences on the cognitive functions, since the abused, besides evident difficulties in total working memory capacity, also has significant difficulties in maintaining attention and verbal information recall. The considerable difficulties of those who have been psychologically abused with attention and verbal memory coincide with biological and clinical premises. In an emotionally threatening situation, such as psychological abuse, the activity of the noradrenergic system, whose lesions cause difficulties in attention, is increased. Furthermore, the secretion of cortisol is increased which further affects changes in the structure of the hippocampus which lies at the foundation of the mentioned functions (Wilson, Hansen, & Li, 2011). From a clinical viewpoint, the repeated psychological abuse of a child is frequently conducted verbally and is tied to several emotional disorders, particularly anxiety (O’Dougherty Wright, Crawford, & Del Castillo, 2009). The effects of emotions on attention have been undoubtedly confirmed, and indicate that anxiety is precisely what limits attention, that is, it reduces attention to the threatening stimuli (Pacheco-Unguetti et al., 2012; Robinson, Krimsky, & Grillon, 2013; Sagaspe, Schwartz, & Vuilleumier, 2011). In persons who have been psychologically abused, the greater part of the cognitive capacity is directed towards “fighting stress” which leads to a division of attention. As a result of insufficiently directed attention, difficulties can arise in the retention of verbal information.

Exposure to maltreatment in childhood, according to the results of this study, correlates positively with all tested working memory capacity functions. The highest correlations which are statistically significant were established for difficulties in working memory verbal information recall and in total working memory amongst persons who had been exposed to psychological abuse and witnessing of family violence. The established connection between psychological abuse and witnessing family violence and difficulties in verbal information recall and in total working memory capacity is in accordance with the earlier described biological changes. Moreover, difficulties in verbal information recall and psychological abuse and witnessing family violence coincide with the foundational assumptions of developmental psychology about the interaction of the environment on the development of verbal functions in children. For example, parents who abuse their children do not encourage verbal expression of the child, and they verbally and non-verbally do not respond to the child’s activity which affects its verbal progress. The obtained results do not support the results of Nikulina and Widom (2013) who found a significant correlation between neglect and poorer executive functioning. The study by Majer et al. (2010) conducted on a sample of 47 adults resulted in significant correlations between psychological abuse and neglect and difficulties in the recall of numerical information. However, as the sample was very small, the obtained results should be considered with caution.

The variables of psychological abuse and witnessing family violence are shown to be the most significant determinants of difficulties in total working memory capacity, verbal information recall and attention ability. In addition to the mentioned difficulties, psychological abuse is a significant predictor for difficulties in executive functioning. The obtained results can be explained as these two types of violence, particularly psychological abuse, represent an especially emotionally traumatic experience for a child, which in the early phase of development affects its neurological development and consequently evokes long-term consequences for the individual (Cook et al., 2005; van der Kolk, 2003).

In conclusion, we may say that persons who have been exposed to maltreatment in childhood have significantly more difficulties in working memory capacity than the participants who never experienced maltreatment. It has also been shown that the difficulties of the abused are manifested in the domain of general working memory capacity and executive functioning. The correlation analysis of the results suggests that all forms of maltreatment are significantly related to all the examined forms of difficulties in working memory capacity, whereby the correlation is highest for total working memory capacity and difficulties in the recall of verbal information. Finally, there is a significant risk for difficulties in working memory capacity amongst those who were exposed to psychological abuse or frequently witnessed family violence. Psychological abuse triggers the greatest negative consequences on cognitive functioning.

### Methodological Limitations of the Study

It is necessary to consider some of the methodological limitations of the conducted study. The heterogeneity of the sample constitutes one of the limitations, as the demographic variables such as age and level of education may have greatly affected the obtained results. The results may have been significantly influenced by the way the abused participants were categorized. According to the models and categorization of exposure to maltreatment in studies to date, arbitrary criteria were determined according to which the abused participants represented a group which was in the upper 25 percentile. A potential deficiency in the selection of such criteria may be the uncertainty in the decision on the correct categorization. Thus, we may have undermined the presence of exposure to maltreatment because we neglected individuals who have been exposed to maltreatment occasionally, but not on a daily basis. A further limitation is the lack of a representative control group. Namely, of the total number of participants, a certain number was classified as a group exposed to maltreatment, and on the basis of assessment, they were sorted into groups according to the type of maltreatment they experienced. It is possible that the participants who were in the group of physically abused were also exposed to other forms of maltreatment besides physical abuse, which could have affected the obtained results.

The applied method for testing exposure to maltreatment was based on the retrospective report of abused persons. As the participants were asked to assess their exposure to maltreatment to a set chronological age, it is possible that some participants forgot or could not recall the events from the past. However, a study conducted on anonymous participants renders it impossible to confirm the responses of the participants through independent sources (Sesar, 2009).

Furthermore, the data which was asked of the participants during the self-assessment of their exposure to maltreatment in childhood are of an intimate nature which could result in arising guilt complexes and thus result in dishonest responses.

It should also be noted that this study did not control the duration, intensity and frequency of the maltreatment, nor the possibility of existing disorders such as post-traumatic stress disorder, attention deficit hyperactivity disorder, depressive disorders and the like, which would greatly decrease the possibility of attributing the obtained results exclusively to maltreatment. To conclude, it is necessary to also address the lack in the implemented questionnaire for testing working memory capacity, as well as the fact that the testing was done electronically. As the method for testing working memory capacity was the method of self-assessment, it is questionable to what extent the participants know themselves, which could have resulted in them supplying answers which do not correspond to their true capacity. Furthermore, the assessments were subjective, which raises the issue of the objectivity of the obtained results.

### Recommendations for Further Research

The results of this research have multiple implications for further research and practice in the field of child maltreatment. This is the first study of its type in the Bosnia and Herzegovina region which comprehensively researched types of violence as determinants of cognitive functioning. The obtained results indicate that different types of maltreatment may lead to different types of cognitive difficulties. One could assume that the type of maltreatment and the duration of maltreatment may increase the susceptibility of neurocognitive dysfunction. Future studies could test how different types of maltreatment in preschool and school age interactively affect the changes in neurocognitive functioning. Furthermore, future studies could conduct a longitudinal study whose aim would be to determine the critical period of the effects of violence on neurobiological development. The measurement of neurobiological changes through brain screening techniques would provide better understanding of the relationship between maltreatment in childhood and cognitive functioning. In addition, future research should use more objective measurements for working memory, as well as clearer criteria with respect to maltreatment. For example, conducting research on a sample of participants who were victims of specific types of abuse in childhood and who were documented by social services would offer a better possibility to generalise the results.
